# Supplementary material for: Deletion of wfs1 Impairs Oligodendrocyte Precursor Cells Dorsal Distribution and Myelination Through the wfs1-hmgcs1 Axis in Zebrafish
Source: Biology (Basel). 2026 Aug 16;15(16):1402. doi: 10.3390/biology15161402 (PMC13510151; doi:10.3390/biology15161402)
Supplement: Supplementary file 1 [file biology-15-01402-s001.zip › File S1-Transcriptome sequencing data and differentially expressed genes (DEGs) in wfs1-- zebrafish at 5 dpf/1.GO/wfs1KOvsWT/GSEA/wfs1KO/GOLGI_ORGANIZATION/GOLGI_ORGANIZATION(GO_0007030).html]

Details for gene set GOLGI\_ORGANIZATION(GO:0007030)[GSEA]


|  || Dataset | deg.sample |
| Phenotype | sample.cls |
| Upregulated in class | wfs1KO |
| GeneSet | GOLGI\_ORGANIZATION(GO:0007030) |
| Enrichment Score (ES) | 0.34814107 |
| Normalized Enrichment Score (NES) | 1.7995993 |
| Nominal p-value | 0.0 |
| FDR q-value | 0.12800759 |
| FWER p-Value | 0.671 |
Table: GSEA Results Summary

  

Fig 1: Enrichment plot: GOLGI\_ORGANIZATION(GO:0007030)
  
Profile of the Running ES Score & Positions of GeneSet Members on the Rank Ordered List

  

| PROBE | DESCRIPTION   (from dataset) | GENE SYMBOL | GENE\_TITLE | RANK IN GENE LIST | RANK METRIC SCORE | RUNNING ES | CORE ENRICHMENT || 1 | 559339 | vps51 |  |  | 433 | 3.166 | 0.0732 | Yes |
| 2 | 553356 | sec16b |  |  | 543 | 2.868 | 0.1485 | Yes |
| 3 | 550341 | dnajc28 |  |  | 689 | 2.616 | 0.2157 | Yes |
| 4 | 393527 | pdcd10a |  |  | 1426 | 1.706 | 0.2394 | Yes |
| 5 | 114452 | blzf1 |  |  | 1825 | 1.448 | 0.2666 | Yes |
| 6 | 450085 | golph3 |  |  | 2088 | 1.330 | 0.2948 | Yes |
| 7 | 566530 | atp8b3 |  |  | 2647 | 1.129 | 0.3083 | Yes |
| 8 | 569617 | golph3l |  |  | 2765 | 1.091 | 0.3345 | Yes |
| 9 | 559291 | atp8b4 |  |  | 3275 | 0.978 | 0.3454 | Yes |
| 10 | 559439 | huwe1 |  |  | 3924 | 0.844 | 0.3481 | Yes |
| 11 | 559941 | arhgef28 |  |  | 5778 | 0.609 | 0.3066 | No |
| 12 | 445093 | tmed1a |  |  | 6513 | 0.536 | 0.2983 | No |
| 13 | 334202 | plk3 |  |  | 6917 | 0.499 | 0.2993 | No |
| 14 | 553061 | cog3 |  |  | 7433 | 0.458 | 0.2957 | No |
| 15 | 570582 | vcpip1 |  |  | 7545 | 0.451 | 0.3046 | No |
| 16 | 101884766 | tmed6 |  |  | 8455 | 0.406 | 0.2871 | No |
| 17 | 492770 | gorasp1a |  |  | 8628 | 0.393 | 0.2925 | No |
| 18 | 336159 | optn |  |  | 9027 | 0.366 | 0.2900 | No |
| 19 | 336789 | vmp1 |  |  | 9113 | 0.359 | 0.2972 | No |
| 20 | 566060 | tjap1 |  |  | 9697 | 0.321 | 0.2877 | No |
| 21 | 100537975 | trip11 |  |  | 10338 | 0.282 | 0.2754 | No |
| 22 | 100537478 | ubxn2a |  |  | 11421 | 0.221 | 0.2474 | No |
| 23 | 100148342 | atp8b1 |  |  | 11709 | 0.205 | 0.2440 | No |
| 24 | 406663 | cog2 |  |  | 12265 | 0.176 | 0.2314 | No |
| 25 | 559775 | lman1 |  |  | 12373 | 0.170 | 0.2327 | No |
| 26 | 559005 | myo18ab |  |  | 12648 | 0.157 | 0.2284 | No |
| 27 | 405848 | tmed7 |  |  | 13394 | 0.121 | 0.2083 | No |
| 28 | 323003 | trappc11 |  |  | 13973 | 0.094 | 0.1928 | No |
| 29 | 492805 | nsfl1c |  |  | 14202 | 0.082 | 0.1878 | No |
| 30 | 393708 | vamp4 |  |  | 14550 | 0.067 | 0.1788 | No |
| 31 | 406667 | pi4k2a |  |  | 14789 | 0.056 | 0.1728 | No |
| 32 | 791158 | tmed1b |  |  | 15022 | 0.045 | 0.1668 | No |
| 33 | 445324 | tango2 |  |  | 15926 | 0.001 | 0.1385 | No |
| 34 | 393676 | gorasp2 |  |  | 16342 | -0.012 | 0.1258 | No |
| 35 | 393591 | zgc:63733 |  |  | 16722 | -0.023 | 0.1145 | No |
| 36 | 566880 | myo18aa |  |  | 17693 | -0.062 | 0.0857 | No |
| 37 | 768247 | cog7 |  |  | 18009 | -0.076 | 0.0779 | No |
| 38 | 393374 | tmed5 |  |  | 18122 | -0.082 | 0.0766 | No |
| 39 | 406694 | vrk1 |  |  | 18325 | -0.090 | 0.0728 | No |
| 40 | 569049 | cog4 |  |  | 18552 | -0.101 | 0.0684 | No |
| 41 | 405869 | pdcd10b |  |  | 18656 | -0.106 | 0.0681 | No |
| 42 | 404604 | clasp2 |  |  | 19579 | -0.147 | 0.0432 | No |
| 43 | 767766 | lman2lb |  |  | 19858 | -0.160 | 0.0388 | No |
| 44 | 557960 | lman2 |  |  | 19993 | -0.166 | 0.0392 | No |
| 45 | 415194 | lysmd3 |  |  | 20475 | -0.186 | 0.0291 | No |
| 46 | 613145 | tmed9 |  |  | 20613 | -0.194 | 0.0302 | No |
| 47 | 572430 | hace1 |  |  | 21796 | -0.256 | 0.0001 | No |
| 48 | 572325 | dym |  |  | 22040 | -0.269 | -0.0002 | No |
| 49 | 564823 | plekhm2 |  |  | 23316 | -0.348 | -0.0307 | No |
| 50 | 321550 | tmed2 |  |  | 24632 | -0.424 | -0.0604 | No |
| 51 | 565431 | golga2 |  |  | 24719 | -0.431 | -0.0512 | No |
| 52 | 798908 | fam174b |  |  | 25167 | -0.457 | -0.0528 | No |
| 53 | 564750 | pi4k2b |  |  | 25560 | -0.490 | -0.0516 | No |
| 54 | 100330945 | atp8b5a |  |  | 25785 | -0.510 | -0.0447 | No |
| 55 | 436746 | arl1 |  |  | 26256 | -0.552 | -0.0443 | No |
| 56 | 406720 | golga5 |  |  | 26597 | -0.587 | -0.0389 | No |
| 57 | 559366 | lrrk2 |  |  | 28112 | -0.758 | -0.0656 | No |
| 58 | 561662 | si:dkey-27b3.2 |  |  | 28802 | -0.879 | -0.0632 | No |
| 59 | 336391 | tmed3 |  |  | 29499 | -1.063 | -0.0559 | No |
| 60 | 415224 | tmed4 |  |  | 29816 | -1.170 | -0.0337 | No |
| 61 | 100126029 | lurap1 |  |  | 31678 | -3.604 | 0.0067 | No |
Table: GSEA details
[plain text format]

  

Fig 2: GOLGI\_ORGANIZATION(GO:0007030)
  
Blue-Pink O' Gram in the Space of the Analyzed GeneSet

  

Fig 3: GOLGI\_ORGANIZATION(GO:0007030): Random ES distribution
  
Gene set null distribution of ES for
 **GOLGI\_ORGANIZATION(GO:0007030)**
